# How tomatoes balance defense: Orchestration of gray mold immunity by the SlMYC2–SlLBD40/42–SlBPM4 module

**DOI:** 10.1093/plcell/koaf261

**Published:** 2025-11-03

**Authors:** Jiajun Wang, Yueyao Wang

**Affiliations:** Assistant Features Editor, The Plant Cell, American Society of Plant Biologists; School of Life Sciences, Xiamen Key Laboratory of Plant Genetics, Xiamen University, Xiamen 361102, China; School of Advanced Agricultural Sciences, Peking University, Beijing 100871, China

*Botrytis cinerea* is a necrotrophic plant fungal pathogen, causing gray mold diseases on many economically important crops. It affects crops at both growing and post-harvest stages, and leading to severe economic losses (Bi et al. 2023). Infection by *B. cinerea* induces the accumulation of jasmonic acid–isoleucine (JA-Ile) in hosts. As an active signaling molecule, JA-Ile binds to the JA receptor COI1, thereby releasing the repression of bHLH transcription factors MYC2 and activating the JA defense signaling pathway (Macioszek et al. 2023). In tomato, SlMYC2 promotes the expression of numerous defense genes and the biosynthesis of secondary metabolites to resist *B. cinerea*, including the ethylene response factor (ERF) ERF.C3, which further activates the key defense gene PR-STH2 involved in plant defense responses (Du et al. 2017).

Lateral organ boundaries domain (LBD) proteins are a plant-specific transcription factor family. In tomato, SlLBD40 and SlMYC2 form a positive feedback regulatory loop to promote fruit enlargement. SlMYC2 activates SlLBD40 transcription, while SlLBD40 stabilizes SlMYC2 protein (Liu et al. 2024). In a new study, **Jialong Zhang and colleagues** (**Zhang et al. 2025**) report that SlLBD40/42 and SlMYC2 establish a negative feedback pathway that prevents tomato immunity from being overactivated during pathogen infection.

Similar to *SlLBD40*, the authors showed that SlMYC2 directly binds to the G-box (CACGTG) in the *SlLBD42* promoter and activates its transcription. In contrast to the *slmyc2* mutant, which is highly susceptible to *B. cinerea*, CRISPR/Cas9 knockout mutants of *SlLBD40* and *SlLBD42* exhibited resistant phenotypes in both leaves and fruits, whereas overexpression lines of *SlLBD40* and *SlLBD42* resulted in susceptibility. Moreover, *sllbd40 slmyc2* and *sllbd42 slmyc2* double mutants remained susceptible but to a lesser extent than *slmyc2*, indicating that SlLBD40/42 antagonize SlMYC2 in regulating tomato resistance to *B. cinerea*.

The authors found that SlLBD40 and SlLBD42 act as transcriptional repressors of defense gene expression, as infection with *B. cinerea* triggered strong upregulation of defense-related genes, including *SlERF.C3*, *SlPR-STH2*, *SlTD*, and *SlJA2L*, in *sllbd40* and *sllbd42* mutants, but the expression of these genes was suppressed in *SlLBD40* and *SlLBD42* overexpression lines. Both SlLBD40 and SlLBD42 contain an EAR (Ethylene-responsive element-binding factor–Associated Amphiphilic Repression) motif at their C terminus, and mutation of this motif completely abolished their transcriptional repression activity. Overexpressing SlLBD40/42 fused to the VP16 transcriptional activation domain exhibited significant resistance to *B. cinerea*, accompanied by significant upregulation of the *B. cinerea*-responsive gene *SlERF.C3*. These results demonstrate that the negative regulation of tomato resistance to *B. cinerea* by SlLBD40/42 is due to their transcriptional repression activity.

To further explore how SlLBD40 and SlLBD42 antagonize SlMYC2 in regulating *B. cinerea* resistance, yeast 1-hybrid, electrophoretic mobility shift, and chromatin immunoprecipitation–qPCR assays revealed that SlLBD40/42 bind to a PBE-box (CACATG) near the start codon of the *SlERF.C3* promoter, whereas SlMYC2 binds to a distal PBE-box. Dual-luciferase reporter assays showed that SlLBD40/42 significantly suppress SlMYC2-mediated activation of the *SlERF.C3* promoter. Consistent with the observation that loss of SlMYC2 compromised the enhanced resistance of the *sllbd40* and *sllbd42* mutants, the upregulation of SlERF.C3 transcripts in these mutants was largely decreased in the *slmyc2* background. These genetic and molecular experiments demonstrate that SlLBD40/42 act to repress SlMYC2-mediated activation of target genes, thereby preventing excessive immune activation. Together with the finding that SlMYC2 positively regulates SlLBD40/42 transcription, these data establish a SlMYC2–SlLBD40/42 negative feedback loop in the regulation of *B. cinerea* resistance.

Through yeast 2-hybrid screening, the BTB/POZ protein SlBPM4 was identified as a SlLBD40/42 interacting protein. The authors confirmed this interaction in the nucleus using bimolecular fluorescence complementation, co-immunoprecipitation, dual-luciferase complementation imaging, and yeast 2-hybrid assays. Protein degradation assays showed that SlLBD40 and SlLBD42 are mainly degraded via the 26S proteasome pathway. In cell-free degradation assays, SlLBD40 and SlLBD42 were degraded significantly more slowly in the *slbpm4* mutant, and their ubiquitination levels were markedly reduced. Similarly, during *B. cinerea* infection, *SlLBD40/42* protein levels accumulated to higher levels in *slbpm4* mutants compared with wild type. Furthermore, when SlLBD40/42 were coexpressed with SlBPM4, their protein levels decreased in a SlBPM4 dose-dependent manner, demonstrating that SlBPM4 functions as an E3 ubiquitin ligase to promote their ubiquitination and degradation. Genetic analyses further showed that *slbpm4* mutants were more susceptible to *B. cinerea*, whereas SlBPM4 overexpression enhanced resistance. *slbpm4 sllbd40* and *slbpm4 sllbd42* double mutants resembled *sllbd40* and *sllbd42* single mutants in showing increased resistance, further supporting the conclusion that SlLBD40/42 act downstream of SlBPM4.

Interestingly, the authors found that upon *B. cinerea* infection, *SlMYC2* transcript levels peaked at 24 h, followed by *SlLBD40* and *SlLBD42* at 36 h, while *SlBPM4* peaked later at around 60 h. Protein accumulation of SlMYC2, SlLBD40/42, and SlBPM4 mirrored this temporal pattern. Therefore, upon *B. cinerea* infection, SlMYC2 activates *SlLBD40/42* transcription, after which SlLBD40/42 suppress SlMYC2-mediated activation of defense genes, acting as brakes to prevent excessive immune activation. As infection progresses, SlBPM4 induction promotes the ubiquitin-dependent degradation of SlLBD40/42 via the 26S proteasome, thereby relieving their repression of SlMYC2 and ensuring a balanced defense response (Fig.).

**Figure.**
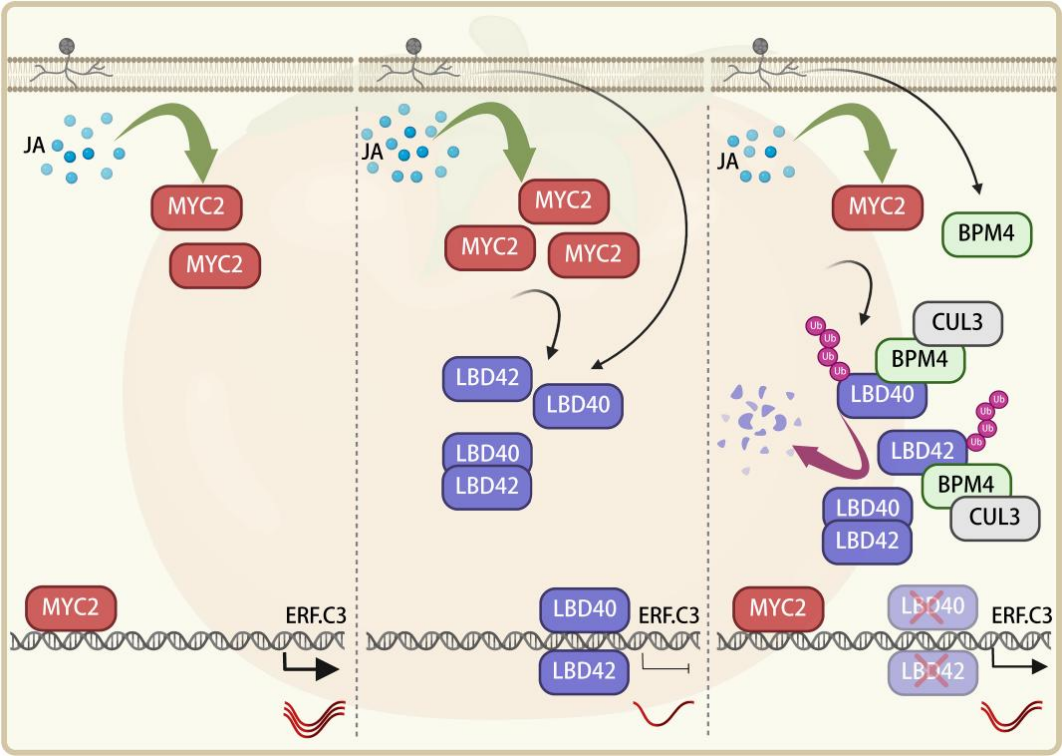
Schematic diagram of the molecular mechanism by which the MYC2-LBD40/42-CRL3^BPM4^ module finely regulates tomato resistance to *B. cinerea*. SlMYC2 acts as the core transcription factor in the JA signaling pathway regulating resistance to *B. cinerea*. During early infection, SlMYC2 promotes the expression of defense genes like *ERF.C3* to combat the pathogen and also promotes increased transcription of *SlLBD40* and *SlLBD42*. SlLBD40 and SlLBD42, in turn, inhibit the transcriptional activation of defense genes by SlMYC2. As the infection progresses, SlBPM4 transcript and protein levels are gradually induced by *B. cinerea*, leading to the ubiquitination and degradation of SlLBD40 and SlLBD42 via the 26S proteasome, thereby relieving their transcriptional repression of SlMYC2. Reprinted from Zhang et al. (2025), Figure 11.

## Recent related articles in *The Plant Cell*:

- Xu et al. (2023) revealed that auxin-induced WRKY23 activates PLT genes while LBD-mediated removal of the repressor bHLH041 derepresses them, and together these coordinated actions establish callus pluripotency in Arabidopsis regeneration.
- Zhang et al. (2025) demonstrated that jasmonate signaling regulates Arabidopsis seed size by coordinating JAZ/MYC-mediated repression with the SOD7-DPA4-KLU pathway, integrating hormonal and transcriptional control to modulate seed growth, especially under salinity stress.
- Wang et al. (2024) showed that jasmonate induces translation of the tRNA-binding protein YUELAO1 via its 3′ UTR, which activates MYC2 and thereby enhances jasmonate responses, revealing a translational layer of regulation in jasmonate signaling.

## Dive Curated Terms

The following phenotypic, genotypic, and functional terms are of significance to the work described in this paper:

SlLBD40 Gramene: Solyc02g085910
SlLBD40 Araport: Solyc02g085910

SlMYC2 Gramene: Solyc08g076930
SlMYC2 Araport: Solyc08g076930

SlLBD42 Gramene: Solyc03g119530
SlLBD42 Araport: Solyc03g119530

SlBPM4 Gramene: Solyc01g009060
SlBPM4 Araport: Solyc01g009060

ERF.C3 Gramene: Solyc09g066360
ERF.C3 Araport: Solyc09g066360
